# Hyaluronic Acid Combined with Serum Rich in Growth Factors in Corneal Epithelial Defects

**DOI:** 10.3390/ijms20071655

**Published:** 2019-04-03

**Authors:** Carlota Suárez-Barrio, Jaime Etxebarria, Raquel Hernáez-Moya, Marina del Val-Alonso, Maddalen Rodriguez-Astigarraga, Arantza Urkaregi, Vanesa Freire, María-Celia Morales, Juan Antonio Durán, Marta Vicario, Irene Molina, Rocío Herrero-Vanrell, Noelia Andollo

**Affiliations:** 1 Department of Cell Biology and Histology, School of Medicine and Nursing, University of the Basque Country, BioCruces Health Research Institute, Begiker, 48940 Leioa, Spain; carlotasb.8@gmail.com (C.S.-B.); JAIME.ECHEVARRIAECENARRO@osakidetza.eus (J.E.); raquel.hernaez@ehu.eus (R.H.-M.); marina_mdv@hotmail.com (M.d.V.-A.); m.rodriguezastigarraga@gmail.com (M.R.-A.); vanesafreire@hotmail.com (V.F.); celiamoralesgonzalez@gmail.com (M.-C.M.); 2 Department of Ophthalmology, University Hospital of Cruces, BioCruces Health Research Institute, Begiker, 48903 Barakaldo, Spain; 3 Department of Applied Mathematics and Statistics and Operational Research, BioCruces Health Research Institute, 48940 Leioa, Spain; arantza.urkaregi@ehu.eus; 4 R & D Department, Instituto Clínico-Quirúrgico de Oftalmología, 48006 Bilbao, Spain; duran@icqo.org; 5 Department of Dermatology, Otorhinolaryngology and Ophthalmology, School of Medicine and Nursing, University of the Basque Country, BioCruces Health Research Institute, Begiker, 48940 Leioa, Spain; 6 Pharmaceutical Innovation in Ophthalmology (InnOftal) UCM Research Group 920415. Department of Pharmaceutics and Food Technology, Faculty of Pharmacy, Complutense University, 28040 Madrid, Spain; mvicario@farm.ucm.es (M.V.); iremm@farm.ucm.es (I.M.); rociohv@farm.ucm.es (R.H.-V.)

**Keywords:** corneal epithelial defect, cornea regeneration, serum eye drops, plasma rich plasma (PRP), serum derived from plasma rich in growth factors (s-PRGF), hyaluronic acid (NaHA), wound healing

## Abstract

The aim of this study is to assess if an adhesive biopolymer, sodium hyaluronate (NaHA), has synergistic effects with s-PRGF (a serum derived from plasma rich in growth factors and a blood derivative that has already shown efficacy in corneal epithelial wound healing), to reduce time of healing or posology. In vitro proliferation and migration studies, both in human corneal epithelial (HCE) cells and in rabbit primary corneal epithelial (RPCE) cultures, were carried out. In addition, we performed studies of corneal wound healing in vivo in rabbits treated with s-PRGF, NaHA, or the combination of both. We performed immunohistochemistry techniques (CK3, CK15, Ki67, ß4 integrin, ZO-1, α-SMA) in rabbit corneas 7 and 30 days after a surgically induced epithelial defect. In vitro results show that the combination of NaHA and s-PRGF offers the worst proliferation rates in both HCE and RPCE cells. Addition of NaHA to s-PRGF diminishes the re-epithelializing capability of s-PRGF. In vivo, all treatments, given twice a day, showed equivalent efficacy in corneal epithelial healing. We conclude that the combined use of s-PRGF and HaNA as an adhesive biopolymer does not improve the efficacy of s-PRGF alone in the wound healing of corneal epithelial defects.

## 1. Introduction

Integrity of the corneal epithelium is a critical requirement for correct vision function [1]. The maintenance of the epithelium is based on a balance among limbal stem function, tear quantity and quality, the eyelid anatomy and function, and corneal sensitivity [2]. In cases of corneal injury, healing mechanisms are activated involving cell proliferation, migration and reattachment of the epithelium to its extracellular matrix, and cell differentiation. Factors needed for corneal wound healing are provided by the tear film, aqueous humor, and limbal blood vessels. Furthermore, cornea epithelium by itself is a rich source of cytokines that contribute to modulate the wound healing process [3]. 

Sometimes, corneal wounds persist over time and are resistant to conventional treatment, such as artificial tears or topical antibiotics [4], lateral tarsorrhaphy [5], bandage contact lenses [6], punctual plugs [7], and amniotic membrane transplantation [8]. Different topical growth factors have been also tested in these persistent epithelial defects [9,10,11,12,13]. As wound healing demands a balanced combination of different mediators, blood derivatives have been used to treat corneal epithelial defects, including autologous serum [14] and platelet rich plasma [15]. One of these, s-PRGF (a serum derived from plasma rich in growth factors) has already been used successfully as a treatment for eye disorders [4,16,17] and its effectiveness has been proved in wound healing [4,18]. s-PRGF has been proved to stimulate proliferation and migration of epithelial cells [18]. It has a moderate platelet concentration and its leukocyte content has been removed [19,20]. 

On the other hand, eye barriers and the continuous turnover of tears can alter the absorption of drugs instilled in the eye, so, although eye drops are an easy-to-use treatment, they must be instilled frequently and/or at high concentrations to achieve therapeutic levels in the tissues. The high frequency of instillation can induce a non-compliance of treatment by patients. The development of vehicles capable of adhering to the conjunctival and/or corneal tissue is an interesting alternative for increasing the bioavailability of ophthalmological medications. With this aim, hydrogels and polymer micelles [21], biodegradable nanocapsules or HA coated nanospheres, and niosomes have been reported as agents for the release of drugs on the ocular surface [22,23,24]. The role of liposomes has also been investigated, although their potential is limited due to their short half-life on the ocular surface and relatively low stability [23]. HA-coated liposomes have also been used to facilitate the entry of drugs into human corneal epithelial (HCE) cells [25]. In all these cases, HA-coated nanovehicles allow greater concentrations of the transported drug to enter into the cornea.

Other authors have tried other vehicles with well-known mucoadhesive properties, with the intention of increasing the contact time of various drugs in the corneal tissue. Thus, the concomitant use of 0.5% carboxymethylcellulose, 0.2% HA, or 0.3–0.5% hydroxypropylcellulose associated with topical 0.5% timolol has been studied. In this case, combination with HA did not show improved efficacy with respect to timolol alone [26]. However, some authors have concluded that an increase of drug viscosity reduces its systemic absorption, so it could enhance the exposition of treatment to the ocular surface [27]. 

Topical surfactant molecules (perfluorohexiloctane), as well as ophthalmic inserts of methylpropylcellulose (Lacrisert^®^), have been developed with the intention of increasing the residence time of the tear on the ocular surface and therein improving the quality [28,29]. Another strategy to prolong the contact of drugs with the ocular surface is the use of contact lenses that slowly release the drug over several weeks [30].

Specifically, to extend the contact time of the platelet lysates with the damaged ocular surface, Sandri and colleagues studied their combination with molecules with mucoadhesive properties, such as polyacrylic acid and chitosan [31]. Similarly, the combination of HA with autologous serum has also been studied, suggesting that HA would facilitate the gradual release of growth factors and increase its duration and effect on the ocular surface, so fewer instillations would be needed [32].

HA is a bioadhesive molecule produced by the cells of the corneal matrix and is one of its main components. It is a polyanionic glycosaminoglycan composed of disaccharide subunits of N-acetyl glucosamine and d-glucuronic acid [33]. Depending on the number of disaccharides bound, hyaluronic acids of different molecular weights will be formed. Among its characteristics, it is noteworthy that it is biocompatible, biodegradable and non-toxic, and non-irritating [34,35]. It also possesses a high capacity for binding to water and has a viscous and pseudoplastic fluid behavior with the ability to act as a mucoadhesive polymer, which makes it possible to increase the residence time in the eye, in addition to reducing friction during blinking and extraocular movements when it is being used as a natural lubricant of the ocular surface that reduces epithelial damage [36]. A negative charge would facilitate adhesion to the ocular surface, giving theoretically more corneal bioavailability to the molecules associated with hyaluronic acid [37]. 

High molecular weight HA has immunosuppressive and anti-inflammatory properties by reducing the migration of inflammatory cells [38] and by specifically inhibiting certain metalloproteases that degrade the extracellular matrix [39]. It also has anti-angiogenic properties [40] and analgesic effects [41]. However, small fragments of HA can have a proinflammatory and pro-angiogenic effect [42]. In our work, we used intermediate molecular weight HA, as we wanted to assess its mucoadhesive capacity for s-PRGF and not its anti-inflammatory synergy. 

All commercial ophthalmic hyaluronic acids used as artificial tears contain concentrations between 0.1% and 0.4% hyaluronic acid. In order to mimic real clinical situations, the concentration used in our work was 0.1% for in vitro assays and 0.2% for in vivo experiments. 

Therefore, the beneficial effect of HA both in vitro and in vivo, as well as Platelet Rich Plasma (PRP), seems to be evident, both in the field of traumatology [43] and in corneal epithelial wound healing [18,44].

Given this “state of the art”, the aim of this study is to test if combining both treatments, s-PRGF and HA, is synergistic in terms of in vitro migration and proliferation of corneal epithelial cells and in vivo reduction of the time (or reduced posology) of corneal wound healing.

## 2. Results

### 2.1. In Vitro Proliferation Assays in Rabbit Primary Corneal Epithelial Cells and Human Corneal Epithlial Cultures

We studied cell proliferation at 0, 24, 48, and 72 h in rabbit epithelial cells (RPCE) and HCE cultures under the following treatments: 45% s-PRGF; 45% s-PRGF + 0.1% sodium hyaluronate (NaHA) (combined treatment); 0.1% NaHA; 10% FBS as a positive/reference control; and 1% BSA as a negative control.

**Figure 1:**
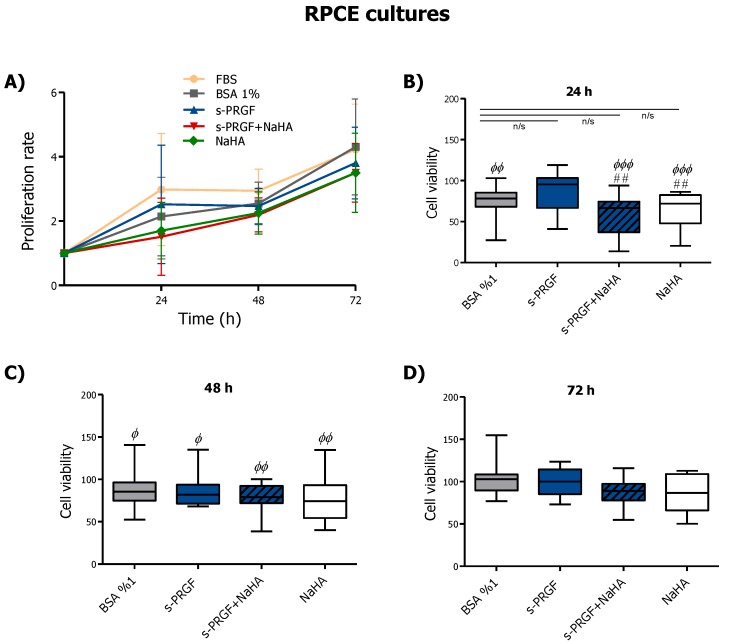
Effect of serum derived from plasma rich in growth factors (s-PRGF), alone or combined with sodium hyaluronate (NaHA), (**A**) on the proliferation and (**B**–**D**) viability of rabbit primary corneal epithelial (RPCE) cultures. Cultures were exposed for 24, 48, and 72 h to 10% FBS; 45% s-PRGF; 45% s-PRGF and 0.1% NaHA (combined treatment); 0.1% NaHA; and 1% BSA as a negative control. Proliferation results are expressed as proliferation rate ± standard deviation of viable cells with respect to viable cells at t = 0. Viability results are expressed as percentages versus that with FBS (100% viability). Statistically significant differences with respect to FBS (Φ) or to s-PRGF (#) (## *p* < 0.01; ^Φ^ *p* < 0.05; ^ΦΦ^ *p* < 0.01; ^ΦΦΦ^ *p* < 0.001; n/s, not significant. Kruskal-Wallis test, Dunn test with Bonferroni correction to Multiple Comparisons).

Results showed that in RPCE cultures all treatments produced a time-dependent proliferation pattern, with no significant differences within treatments at 72 h (Figure 1A). Viability in RPCE cultures exposed to different treatments was very similar in all cases. We observed that viability in the first 24–48 h (Figure 1B,C) was higher with FBS, the standard or reference culture medium. However, differences decreased over time, especially under s-PRGF and control (BSA) treatments. Thus, after 24 h of treatment, we observed highly significant differences between FBS and NaHA, alone or combined with s-PRGF, and between FBS and the control treatment (Figure 1B). However, there were not significant differences between FBS and s-PRGF at 24 h (*p* = 0.42). In addition, we found significant differences in cell viability between cells cultured with s-PRGF in comparison to those cultured with both NaHA treatments. 

Thus, proliferation of RPCE cultures at 24 h was similar for FBS and s-PRGF treated cultures and higher than cultures under the other treatments. At 48 h, we found significant differences within treatments compared to FBS, with these being less than those at 24 h and completely disappearing at 72 h (Figure 1B–D).

In summary, NaHA, whether combined or not with s-PRGF, did not enhance either proliferation capability or viability in RPCE cultures.

**Figure 2:**
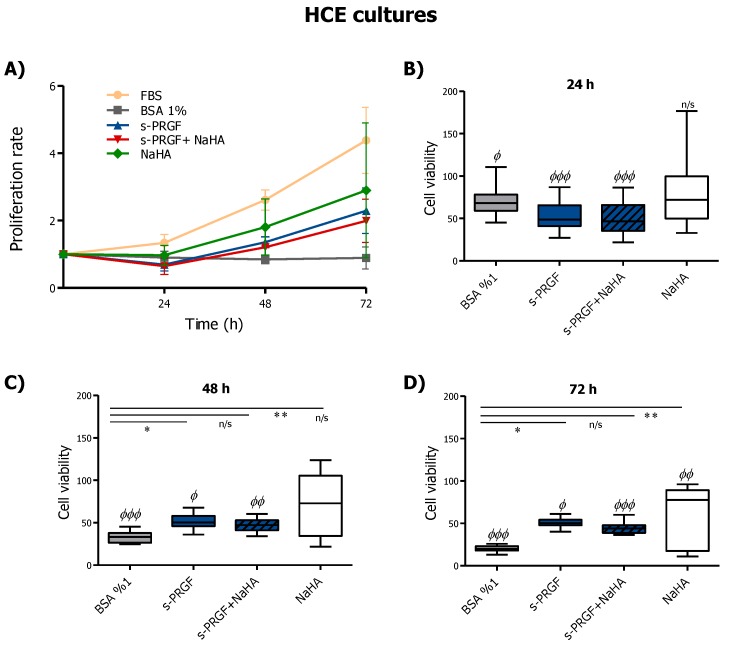
Effect of s-PRGF, alone or combined with NaHA, on the (**A**) proliferation and (**B**–**D**) viability of human corneal epithelial (HCE) cells. Cultures were exposed for 24, 48, and 72 h to 10% FBS; 45% s-PRGF; 45% s-PRGF and 0.1% NaHA (combined treatment); 0.1% NaHA; and 1% BSA as a negative control. Proliferation results are expressed as proliferation rate ± standard deviation of viable cells with respect to viable cells at t = 0. Viability results are expressed as percentages versus that with FBS (100% viability). Statistically significant differences with respect to BSA (*) or to FBS (Φ) (* *p* < 0.05; ** *p* < 0.01; ^Φ^ *p* < 0.05; ^ΦΦ^ *p* < 0.01; ^ΦΦΦ^ *p* < 0.001; n/s, not significant. Kruskal–Wallis test, Dunn test with Bonferroni correction to Multiple Comparisons).

Results concerning HCE cultures showed a time-dependent proliferation pattern, except for the 1% BSA control treatment, while s-PRGF, with or without NaHA, produced a decrease in proliferation at 24 h which was not statistically significant (Figure 2A). All treatments, especially 10% FBS, showed a higher proliferation rate that the control treatment. In addition, we saw a positive tendency for higher proliferation when cells were cultured with NaHA compared to s-PRGF or the combined treatment.

Regarding viability, we do not see significant differences between FBS and NaHA during the first 48 h (Figure 2B,C). In addition, besides the FBS treatment, only the non-combined treatments showed significant (s-PRGF) or very significant (NaHA) differences compared to the control treatment at 48 and 72 h (Figure 2C,D). 

In conclusion, s-PRGF and NaHA treatments showed better proliferative patterns in HCE cells, whereas the combination of both did not improve it.

### 2.2. In Vitro Scratch Wound-Healing Assays in RPCE and HCE Cultures

In order to evaluate the capability of the different treatments to promote migration and re-epithelialization on RCPE and HCE cultures, we scraped off rounded areas on cell monolayers and treated them with the following treatments: 45% s-PRGF; 45% s-PRGF + 0,1% NaHA (combined treatment); 0,1% NaHA; 10% FBS as a positive/reference control; and 1% BSA as a negative control. We measured the re-epithelialization process at 0, 12, 24, 36, 48, 60, and 72 h.

**Figure 3:**
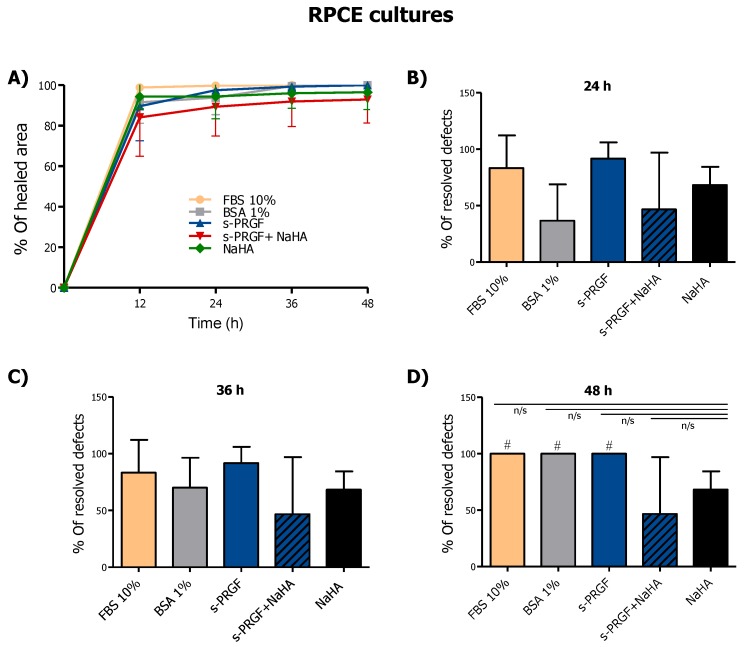
Effect of s-PRGF, alone or combined with NaHA, on the re-epithelialization of rabbit primary corneal epithelial (RPCE) cultures. Cultures were exposed for 72 h to 10% FBS; 45% s-PRGF; 45% s-PRGF + 0.1% NaHA (combined treatment); 0.1% NaHA; and 1% BSA as a negative control. The percentage of re-epithelialized area of RPCE cultures after 48 hr (**A**), and percentage of wells in which the defect in the monolayer had completely resolved at 24 (**B**), 36 (**C**) and 48 (**D**) hours are shown. Statistically significant differences with respect to the combined s-PRGF + NaHA treatment (#) (# *p* < 0.05; n/s, not significant. χ^2^ test and Fisher’s exact test).

We did not find significant differences within treatments at any time when studying wound healing evolution in RPCE cultures (Figure 3A). However, when we analyzed the percentage of wells in which the defect in the monolayer had completely resolved, we found evident differences in cultures treated with NaHA (alone or combined) with respect to other treatments (Figure 3B–D). Additionally, a smaller number of completely resolved defects in cultures treated with the combined treatment was observed, with this result being statistically significant from 48 h. 

**Figure 4:**
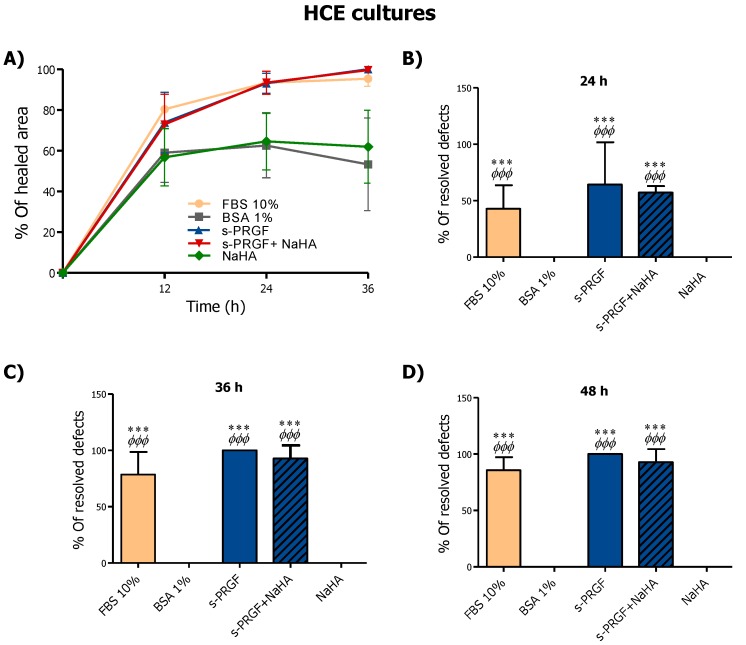
Effect of s-PRGF, alone or combined with NaHA, on the re-epithelialization of human corneal epithelial (HCE) cells (**A**). Cultures were exposed for 72 h to 10% FBS; 45% s-PRGF; 45% s-PRGF + 0.1% NaHA (combined treatment); 0.1% NaHA; and 1% BSA as a negative control. The percentage of re-epithelialized area of HCE cultures after 36 hr (**A**), and percentage of wells in which the defect in the monolayer had completely resolved at 24 (**B**), 36 (**C**) and 48 (**D**) hours are shown. Statistically significant differences with respect to BSA (*) or NaHA (Φ) (*** *p* < 0.001; ^ΦΦΦ^ *p* < 0.001; n/s, not significant. χ^2^ test and Fisher’s exact test).

In HCE cultures, s-PRGF treatment (alone or combined with NaHA), as well as FBS treatment, promoted faster re-epithelialization of the defect in the monolayer from 12 h of treatment onwards, showing significant differences at all times (Figure 4A). Furthermore, in the HCE cultures, no statistically significant differences were found in the mean remaining denuded area (in square millimeters) between cells treated with NaHA and control cells.

With respect to the resolution of defects in the HCE cultures, s-PRGF treatment (alone or combined), together with FBS, produced statistically significant differences in the number of wells in which the denuded area had been completely covered, compared with the control and NaHA treatments, from 24 h (Figure 4B). At 36 h, almost 100% of wounds treated with s-PRGF or s-PRGF + NaHA were completely solved, whereas none of the denuded areas had completely closed in the control and NaHA cultures (Figure 4C,D). 

We conclude that s-PRGF promotes the highest re-epithelialization in RPCE primary cultures and HCE cells. Furthermore, NaHA does not favor this process, and hinders the re-epithelialization effect promoted by s-PRGF.

### 2.3. In Vivo Corneal Re-Epithelialization Assay in a Rabbit Animal Model

To perform the assay, surgically induced epithelial defects were treated with 90% s-PRGF; 90% s-PRGF and 0.2% NaHA (combined treatment); 0.2% NaHA; and PBS as a control treatment.

We did not find any adverse effects, such as corneal inflammation or neovascularization, during the whole experiment. In addition, all animals were healthy and gained weight progressively. 

**Figure 5:**
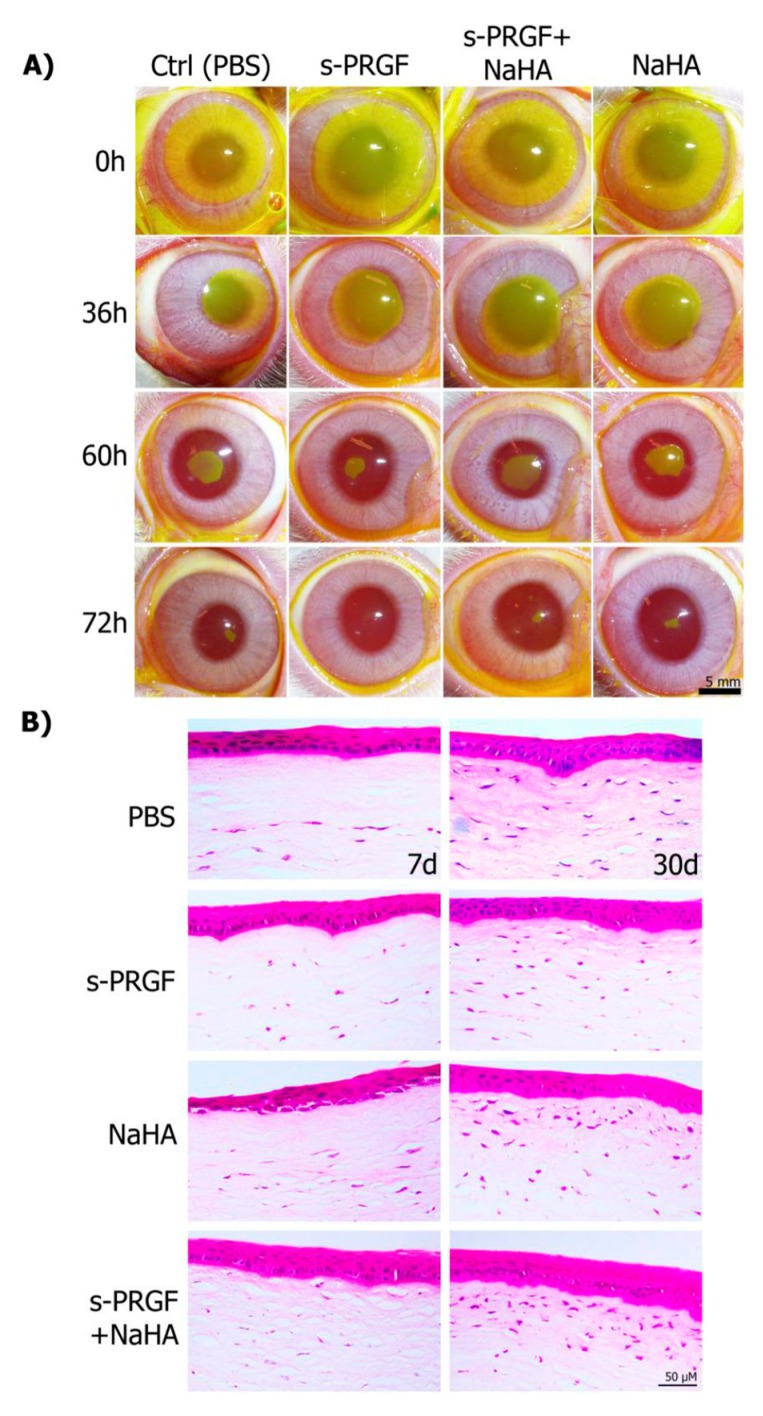
Re-epithelialization of corneal defects in rabbit eyes. (**A**) Evolution of the epithelial defect was monitored with fluorescein staining in eyes treated with topical s-PRGF, s-PRGF and NaHA, NaHA, or PBS as a control treatment. (**B**) Histological sections of rabbit central corneas after complete healing were stained with hematoxylin and eosin. Corneas were processed seven days after surgery or at 30 days. Scale bar: 5 mm for (**A**), 50 µm for (**B**).

The results showed that s-PRGF promoted faster corneal wound healing after day 2 of treatment than the other treatments. The mean time to complete the closure of the epithelial defect in the s-PRGF group was 3.11 ± 0.22 days, whereas it was 3.31 ± 0.37 days for eyes treated with any of the other treatments (Figure 5A and Table 1). Nevertheless, we did not find significant differences among treatments (Kruskal–Wallis). We also performed the Kaplan–Meier test to analyze the progression of healing of eyes at intervals of half a day. Although we again observed the marked tendency for faster epithelial closure for the s-PRGF treatment, we could not find significant differences. This fact suggests that increasing the number of analyzed animals would be advisable.

When we analyzed the number of corneal defects that had completely healed, we found that at day 3 after surgery, 78% of them had re-epithelialized in the s-PRGF treatment, while only 50% of them had healed in the eyes treated with any of the other treatments. After 3.5 days, 100% of the corneal defects had already healed with s-PRGF, compared to only 88% of the eyes treated with any of the other treatments. However, we did not find significant differences among treatments (Chi-Square test and Fisher’s exact test).

Analysis of hematoxylin and eosin sections of the rabbit central corneas showed complete regeneration with normal histology of the epithelium in all corneas. However, we observed that corneas treated with NaHA showed a less compacted epithelium in the basal layers at day 7, suggesting that there might be adhesion deficiencies within epithelial layers, or even between the epithelium and stroma layers (Figure 5B). In addition, when euthanasia was performed 7 days after surgery, the number of keratocytes in the anterior third of the stroma was influenced by the treatment, with the s-PRGF and NaHA the only treatment that showed cells in the whole stroma. At 30 days after surgery, treatments with NaHA (alone or combined) showed the highest cell population across the anterior third of the stroma. 

### 2.4. Immunohistochemical Analyses of the Epithelial Differentiation, Proliferation, Adhesion, and Fibrosis of the Re-Epithelialized Corneas

To assess differences in the mechanisms through which the treatments performed corneal wound healing, we used immunohistochemistry techniques to analyze cryopreserved sections of healed rabbit corneas at 7 and 30 days after surgery. We also added a healthy control (healthy rabbit cornea, which did not undergo surgery) and a wounded cornea (processed only 48 h after surgery, wound healing or W-H control). Specifically, we studied the processes of differentiation, proliferation and adhesion, the corneal barrier effect of the epithelium, and stromal fibrosis.

**Figure 6:**
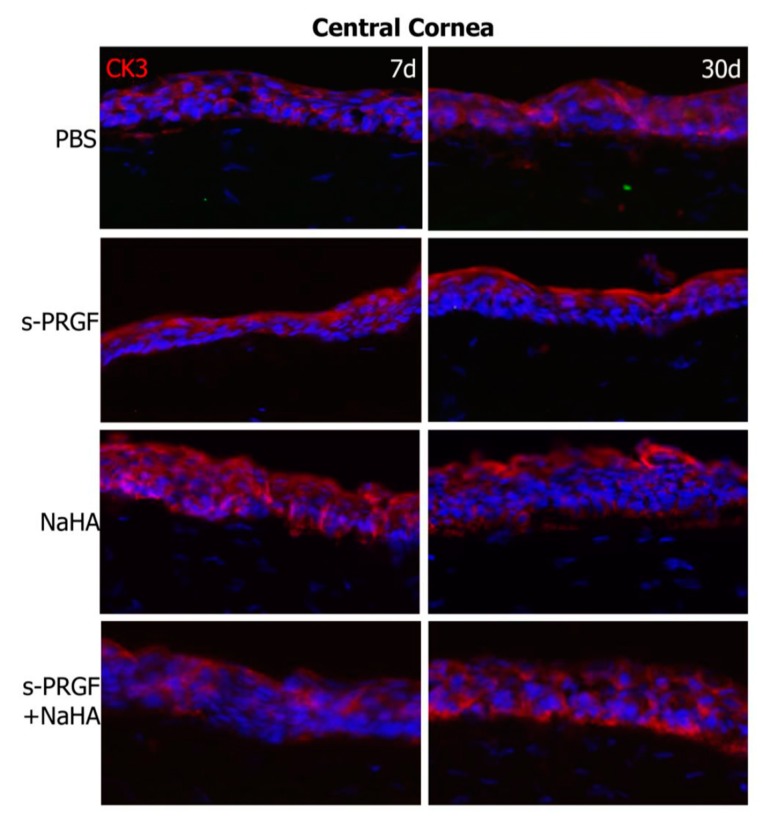
Fluorescent immunostaining for CK3 (red) and CK15 (green) on the regenerated central cornea of rabbit corneas after healing of the epithelial defect. Corneas were treated with s-PRGF, s-PRGF and NaHA, NaHA, or PBS (as a control) and were processed 7 and 30 days after cornea surgery. Control corresponds to a healthy rabbit cornea with no surgery. The W-H image shows a cornea processed 48 h after surgery without complete re-epithelialization. Magnification 200×.

**Figure 7:**
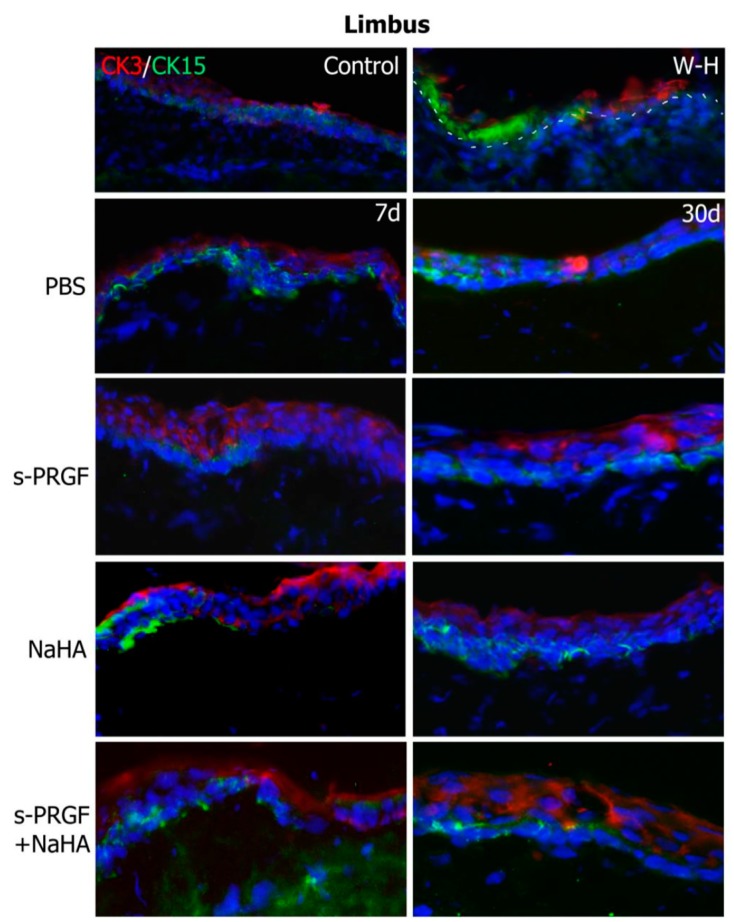
Fluorescent immunostaining for CK3 (red) and CK15 (green) on the limbal area of rabbit corneas after healing of the epithelial defect. Corneas were treated with s-PRGF, s-PRGF and NaHA, NaHA, or PBS (as a control) and were processed 7 and 30 days after cornea surgery. Control corresponds to a healthy rabbit cornea with no surgery. The W-H image shows a cornea processed 48 h after surgery without complete re-epithelialization. The dotted line shows the limit between the epithelium and stroma layers. Magnification 200×.

First, we performed a double staining for cytokeratin 3/76 (CK3), a corneal epithelium marker, and cytokeratin 15 (CK15), a stem cell marker, in both the re-epithelialized central cornea and the peripheral limbus. As we expected, we found CK3 positive staining and CK15 negative labeling in the central epithelium area of all eyes (Figure 6). At the limbal area, we found positive CK15 staining in the basal layers of all corneas (Figure 7). This staining is coherent with the presence of limbal stem/progenitor cells, and it was especially intense in the W-H control, where these cells might be specifically activated to regenerate the wounded epithelial area.

We evaluated cell proliferation by analyzing the nuclear staining of the proliferation marker Ki76. Results showed a higher number of positive cells at 7 days after surgery than 30 days (Table 2). Interestingly, at 7 days, the nuclear staining was not confined to the epithelium but also appeared in the third anterior stroma. However, proliferation in the epithelium was significantly higher than in the stroma for all treatments, both at 7 days (*p* < 0.001) and at 30 days (*p* < 0.0001) (Wilcoxon rank sum test). Specifically, corneas treated with NaHA showed a higher number of Ki67 proliferative cells in both areas (epithelium and stroma) than corneas under the other treatments. Differences were highly significant for the epithelium (Table 2). This result is consistent with that of the proliferation study in HCE cells, in which we observed that NaHA induced higher proliferation in the short-term than the rest of the treatments under study. At 30 days after surgery, cell proliferation was lower and occurred merely in the corneal epithelium.

**Figure 8:**
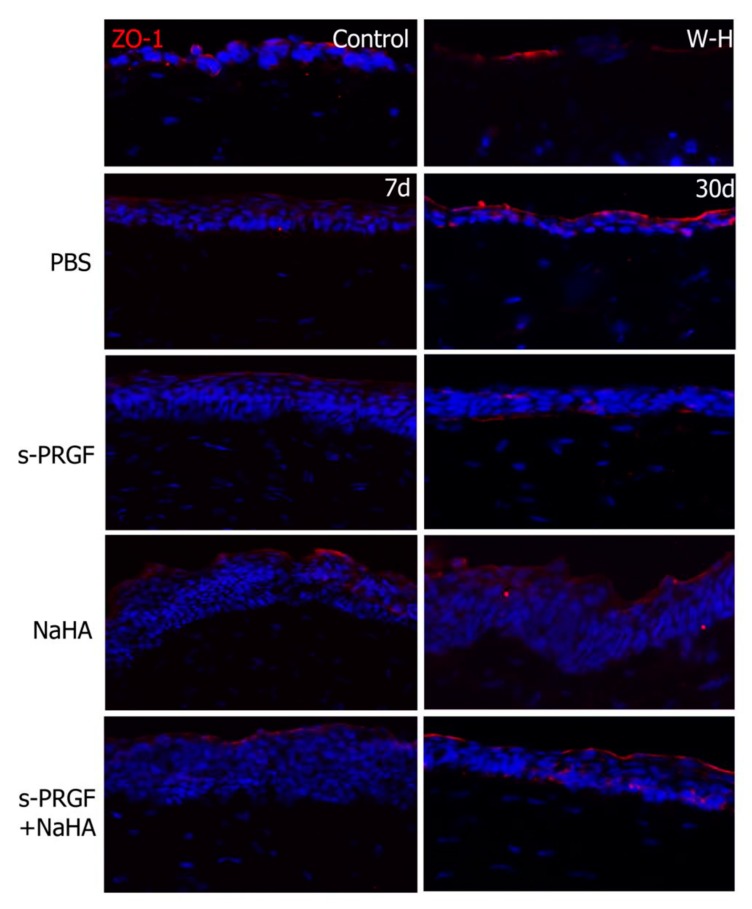
Fluorescent immunostaining for ZO-1 on rabbit central corneas after healing of the epithelial defect. Rabbit corneas were treated with s-PRGF, s-PRGF and NaHA, NaHA, or PBS (as a control), and were processed 7 and 30 days after cornea surgery. Control corresponds to a healthy rabbit cornea with no surgery. The W-H image shows a cornea processed 48 h after surgery without complete re-epithelialization. Magnification 200×.

We also studied the marker of tight junctions ZO-1, in order to evaluate the recovery of the barrier effect in the regenerated corneal epithelium. We observed apical staining of the epithelium in all corneas, which was more intense at 30 days after surgery than at 7 days (Figure 8). Of note, W-H corneas, which only had a cell monolayer covering the wound area, showed a positive staining as well, meaning that the recovering of the epithelial barrier function is a priority in wound healing. 

**Figure 9:**
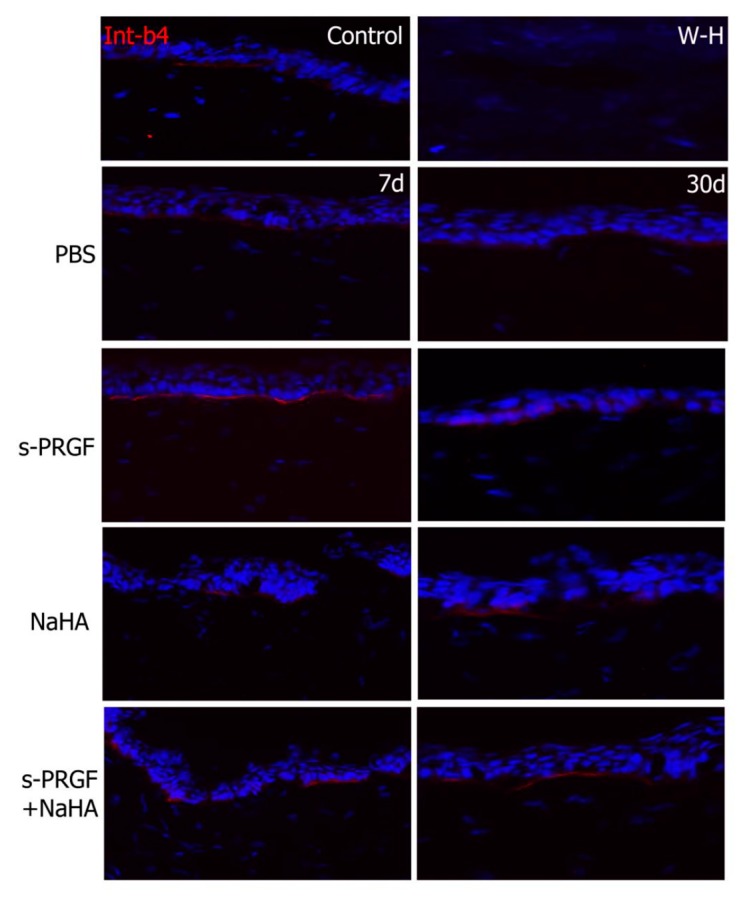
Fluorescent immunostaining for β4 integrin on rabbit central corneas after healing of the epithelial defect. Rabbit corneas were treated with s-PRGF, s-PRGF and NaHA, NaHA, or PBS (as a control) and were processed 7 and 30 days after cornea surgery. Control corresponds to a healthy rabbit cornea with no surgery. The W-H image shows a cornea processed 48 h after surgery without complete re-epithelialization. Magnification 200×.

In order to study the adhesion property between the regenerated epithelium and the underlying stroma, we performed immunohistochemical staining for β4 integrin, a cellular component of hemidesmosomes. Results showed that s-PRGF treatments (combined or not) had a more intense and continuous staining at 7 days (Figure 9). At 30 days after surgery, we could observe a normal staining underlying the epithelium all along the cornea in all the treatments. In W-H samples, although a fine layer of regenerated epithelium appeared, we could not detect the β4 integrin staining.

Finally, by detecting the α-SMA protein, we analyzed the differentiation process from keratocytes to myofibroblasts in the wounded area as a sign of fibrosis. We did not detect positivity in the cytoplasm of the stromal cells of the repaired tissues in any case (data not shown). 

## 3. Discussion

It has been shown that HA improves in vitro proliferation and migration of corneal and conjunctival epithelium [44,45]. Moreover, it stabilizes the epithelial barrier of the corneal surface by binding to its corneal and conjunctiva receptor, hialadherin CD44 [46]. It has been also proven that HA helps migration and proliferation of fibroblasts [27]. In addition, it has no cytotoxicity to epithelial cells of the ocular surface, has antioxidant properties, and tends to reduce the toxic effects of preservatives [47]. However, other studies have indicated that HA specifically influences the migration of corneal epithelial cells, but not the proliferation, so that the benefit of HA in the healing of corneal wounds would be related to rapid cell migration [48]. 

Taking into account these results and based on the premise that HA has bioadhesive properties, we set out to assess whether the combination of the blood product s-PRGF with HA was able to increase the exposure time of the blood product to the cornea, so that the number of instillations in the treatment of the corneal epithelial defect could be reduced, thus facilitating therapeutic compliance in the case of clinical treatments. To this aim, we performed in vitro proliferation and migration assays, as well as in vivo assays in a rabbit model of an induced corneal epithelial defect. 

Our proliferation results show that HA (in our case, sodium hyaluronate or NaHA) alone favors the proliferation of the human corneal epithelial line HCE, with better results than s-PRGF and the combination of both. However, the effect of NaHA on the capacity of induction of proliferation on the rabbit primary cells RPCE is similar to that of the other treatments, including the reference treatment with FBS and the negative control (BSA). On the other hand, the analysis of the Ki67 proliferation marker confirms a greater proliferative effect of NaHA on the central corneal epithelial cells (as well as on stromal keratocytes), with respect to the rest of treatments. This difference in proliferation rates according to the cell type studied may be due to the fact that the HCE are more differentiated cells and are more sensitive to certain signals in the microenvironment, while the RPCE are cells with greater intrinsic power since they contain progenitor epithelial cells. According to certain authors, the ability to stimulate proliferation on corneal epithelial cells depends on the concentration of EGF in the medium they are cultured with [13]. We observed, in the primary cultures, equivalent proliferation rates with medium containing EGF (s-PRGF treatment, combined or not combined with NaHa), or not containing EGF (NaHA and even in the BSA control treatment). Therefore, we propose that the proliferation of RPCE cells is independent, in the short term, from the medium to which they are exposed, since they have an intrinsic proliferation capacity. However, HCE epithelial cells mimic cells in the central cornea and show increased proliferation in response to HA.

On the other hand, our results suggest that a negative interaction between s-PRGF and NaHA occurs, so that the proliferative capacity of the HCE cells decreases when both are combined with respect to any of them alone. This result is supported by studies by other authors who have tested other mucoadhesive polymers, such as polyacrilic acid, combined with the platelet lysate in rabbit primary cultures, in both corneal epithelial cells and keratocytes. They show that cell proliferation is lower than when using platelet lysate alone [31]. 

However, the combination of HA with PRP on chondrocytes (mesodermal cells, such as corneal fibroblasts) improved cell proliferation, although not in a statistically significant manner [49], while significant changes in the expression levels of certain inflammatory markers and extracellular matrix proteins were observed. In addition, randomized controlled studies showed improvement in the clinical outcome of osteoarthritis of the knee when treated with HA and PRP with respect to PRP at three months, as well as with respect to HA at one year [50].

Considering this, we can conclude that the combination of s-PRGF and NaHA does not act synergistically in the proliferation of some kinds of cells of ectodermal origin, such as the corneal epithelium, and could even disadvantage the proliferative effect that HA has. Conversely, this combination does favor the proliferation of some kinds of mesodermal cells [43,49]. All of this suggests that the effect of the PRPs or the HA, or the combination of both, is dependent on the cell type and that it can also favor different biological functions depending on the case. 

To study the effect of the combination of NaHA and s-PRGF on corneal re-epithelialization, we performed an in vitro wound healing assay in HCE cells and RPCE cells. We observed that the NaHA alone is not as good as s-PRGF alone for re-epithelialization. In fact, s-PRGF alone is the treatment that best stimulates corneal re-epithelialization. However, NaHA alone stimulates cell migration to a greater extent in primary cultures (RPCE) than in the HCE cell line. On the other hand, the combination of both did not provide any benefit in most of the assays, or was even counterproductive in the in vitro test with RPCE, in which the addition of NaHA to the blood product impairs the re-epithelialization capacity of s-PRGF. 

The fact that NaHA favors, in a certain way, the epithelial closure of the RPCE could be explained by the fact that HA is an essential component of the niche matrix of limbal progenitor cells [51], so that more undifferentiated cells, such as the RPCE (which still retain the HA receptors), when in contact with HA, could migrate more actively than differentiated corneal epithelial cells (HCE).

We did not observe significant differences in the in vivo re-epithelialization capacity between any of the treatments used (s-PRGF, NaHA, both combined, or PBS as the control treatment), although s-PRGF offered a tendency to achieve better results both in the evolution of the area of the corneal defect and in the number of defects totally closed at certain times. In other in vivo studies performed on mesodermal tissues, different results have been found: Cartilage repair of better quality is achieved when PRP is combined with HA, compared to HA alone [52], and excellent results with the combination of HA and PRP in the repair of pressure ulcers and surgical wounds have also been reported [53]. However, other authors have shown equal cartilage repair capacity histologically in an in vivo model when using PRP, with respect to the combined use of PRP and HA [54].

Our histological analysis by hematoxylin and eosin staining showed correct epithelia in all cases, although those treated with NaHA were more disorganized than those undergoing other treatments or healthy controls. In addition, a greater density of fibroblast cells was observed in the anterior stroma of the corneas treated with NaHA, alone or combined with s-PRGF, at 30 days after in vivo scraping surgery. It has been described that stromal cells, possibly myofibroblasts, migrate to the superficial layers of the stroma to help close the exposed area [55]. By Ki67 cell proliferation labeling, we have demonstrated that NaHA induces proliferation in both epithelial and stromal cells, mainly in the first days after injury. The rapid initial epithelial proliferation may be the reason for the disorganization of the epithelia in the corneas under this treatment. However, the proliferation (Ki67 positive cells) obtained in the anterior stroma suggests that the accumulation of cells in that area is not only due to the cell proliferation process, but is also the result of cell migration, as other authors have described. In the case of combined treatment, s-PRGF can also contribute to this, since the histological images show the highest cell density for this treatment. In addition, we have already demonstrated through transwell-type migration experiments that s-PRGF exerts a chemotactic effect on corneal keratocytes [18].

The immunohistochemical analysis revealed similar results for the CK3 and CK15 markers, as well as for the ZO-1 protein, between the different treatments. CK15 is a marker of progenitor cells of the corneal epithelium [56], whose positivity is restricted to the basal layers of the limbal epithelium. Our results confirm the presence of these cells in the sclero-corneal limbus of all the corneas studied. When these cells divide and differentiate towards corneal epithelial cells, during their displacement of centripetal and ascending form in the cornea, they progressively express a greater labeling of the CK3 [57,58]. Similarly, the epithelial cells located in the most anterior part of the corneal epithelium express the protein belonging to the tight junction ZO-1 [59]. In all treatments, both at 7 days and 30 days after surgery, we observed a similar labeling of both proteins, demonstrating that all treatments achieve re-epithelialization of damaged corneas, generating a mature and functional corneal epithelium. It is curious to observe how the barrier function of the corneal epithelium (positivity for ZO-1) is established from the very beginning of the repair of the lesion, being observed even in those incipient epithelia formed by a single cell monolayer. Therefore, we could suggest that the establishment of the corneal barrier function is a priority in corneal healing.

Regarding the adhesion property between the newly repaired epithelium and the underlying extracellular matrix, our results show that it could be favored by treatment with s-PRGF. Thus, the β4 integrin protein is one of the components that forms part of the hemidesmosome-type junctions between the epithelium and the matrix [60], in order to achieve a compact and stable tissue. At short follow up times (7 days post-surgery), corneas treated with s-PRGF, alone or in combination, show the most intense and continuous labeling for β4 integrin, suggesting that this hemoderivative favors epithelial adhesion and that the combination with NaHA does not diminish this effect. This data is very important, since it can explain the efficacy of s-PRGF in the treatment of persistent and recurrent corneal epithelial defects [4].

## 4. Materials and Methods

### 4.1. Ethics Statements

This study was performed in accordance with the ARVO Statement for the Use of Animals in Ophthalmic and Vision Research. The procedures and experimental designs were approved by the Animal Experimentation Ethics Committee of the University of the Basque Country UPV/EHU (Permit license: CEBA/49-P03-02/2010/ANDOLLO VICTORIANO, 2011/03/11) and fulfill European and national laws.

### 4.2. Isolation and Expansion of Rabbit Primary Corneal Epithelial (RPCE) Cultures

To obtain RPCE cultures, 0.5 × 0.3 cm explants of corneas, including the limbal area, were seeded in plastic culture wells, with the corneal stroma down. We used corneas from the eyes of three 2.0–2.5 kg female New Zealand rabbits. The cells that grew from the explants were maintained at 37 °C under 5% CO_2_ in DMEM: Ham′s F12 mix with 2 mM L-glutamine (Lonza, Verviers, Belgium) and 1% penicillin–streptomycin (Lonza), together with 10% fetal bovine serum (FBS; Lonza). This culture medium was also supplemented with 10 ng/mL EGF (Sigma, St. Louis, MO, USA), 5 μg/mL insulin (Sigma), and 0.1 μg/mL cholera toxin (Gentaur Molecular Products, Brussels, Belgium). The positive staining for corneal epithelial markers was confirmed by immunolabeling. Cells were positive for the CK3 corneal epithelial, as well as for the CK15 and vimentin corneal epithelial stem/progenitor cell markers (Data not shown or Figure S1).

### 4.3. Human Corneal Epithelial (HCE) Cell Line Culture

SV-40 immortalized HCE cells were kindly provided by Dr. Araki-Sasaki et al. These cells were cultured at 37 °C under the same conditions as the RPCE cells, with the addition of the supplement 0.5% DMSO (Sigma) to the culture medium.

### 4.4. s-PRGF Preparation

For human s-PRGF preparation, blood was collected by venipuncture in tubes with 3.8% sodium citrate as an anticoagulant (BD Biosciences, Franklin Lakes, NJ, USA). Blood was centrifuged for 8 min at 460× *g*. After collection of the complete supernatant fraction above the buffy coat, in order to induce clot formation, calcium chloride (Braun, Barcelona, Spain) was added at a final concentration of 22.8 mM. After incubation of the samples for 2 h at 36 °C, the fibrin clot was retracted and removed; the remaining fraction was the s-PRGF [61].

For rabbit s-PRGF preparation, the human protocol varied as follows: Blood was centrifuged for 8 min at 650 g. Following addition of calcium chloride, samples were incubated for 1 h at 36 °C [18].

For in vitro assays, the complement was heat inactivated in s-PRGF, and samples from several individuals pooled to obtain representative blood preparations that could provide reproducible results with minimal interindividual variability. Samples were stored at −20 °C. 

For in vivo assays, autologous s-PRGF was obtained and stored at −20 °C until use.

### 4.5. Bioadhesive (Hyaluronic Acid) Preparation

We used an ophthalmic grade sodium hyaluronate (NaHA) with a molecular weight of 200–400 kDa (Abarán Materias Primas SL, Barcelona, Spain) in PBS (phosphate buffered saline solution). 

### 4.6. In Vitro and In Vivo Treatments

RPCE and HCE cells were cultured under the following treatments for in vitro experiments: 1% BSA (Bovine serum albumin); 10% FBS; 45% s-PRGF; 45% s-PRGF and 0.1% NaHA; and 0.1% NaHA. The above products were diluted in supplemented culture medium (Table S1). 

We used the following treatments for in vivo experiments: PBS; 90% s-PRGF; 90% s-PRGF and 0.2% NaHA; and 0.2% NaHA. The above products were diluted in PBS.

### 4.7. Cell Proliferation Assays

For these experiments, 3000 HCE cells (2500 in the case of RPCE cells) were seeded per well in 96-well plates. After synchronizing the cultures using DMEM:F12 with 1% BSA (Table S1) for 16 h, culture medium was substituted by the treatments to be tested (Table S1). Cell proliferation was analyzed at 0, 24, 48, and 72 h. Proliferation was described in terms of proliferation rate ± SD of viable cells, with respect to viable cells just before exposure to treatment (t = 0 h). This was measured using a 3-[4,5-dimethylthiazol-2-yl]-2,5-diphenyl tetrazolium bromide or MTT assay (Sigma-Aldrich), as previously described [61]. Optical densities at 540 nm were determined using a microplate reader (ELx800 Microplate Reader, BioTek^®^ Instruments, Winooski, VT, USA). All experiments were performed in quadruplicate and repeated in three biological replicates.

### 4.8. In Vitro Wound Healing Assays

These experiments were performed as previously described [1]. Briefly, 25,000 RPCE culture cells (20,000 in the case of HCE cells) were seeded per well in 96-well plates and left to form monolayers. Once the cultures were synchronized by using DMEM:F12 with 1% BSA (Table S1) for 16 h, a circular central epithelial defect was created using a tip. Wells were divided in groups depending on the treatment (Table S1). After that, areas from which cells had been scraped away were photographed every 12 h with a phase contrast microscope (Nikon Eclipse TS 100; Nikon, Tokyo, Japan) and the images were acquired with the ProgRes CapturePro 2.6 software (Jenoptik, Jena, Germany). The size of the denuded areas was quantified using ImageJ software (developed by Wayne Rasband at the Research Services Branch, National Institute of Mental Health, Bethesda, MD). The closure rate was described in terms of the mean remaining denuded area ± SD in square millimeters. All the experiments were performed at least in quintuplicate (5 wells) and repeated in three biological replicates of RPCE and HCE cultures. For wound healing experiments in HCE cells (with high proliferation capacity), cells were previously treated with 10 μg/mL of mitomycin C for 3 h at 37 °C and washed three times with PBS after that. Afterwards, HCE cells were separated from the well by using EDTA-trypsin, and then seeded. In the case of RPCE cells, this step was not needed.

### 4.9. In Vivo Rabbit Corneal Re-Epithelialization Assays

Seventeen adult 2.0–2.5 kg female New Zealand white rabbits (33 eyes) were included in the study. They were under diary observation to assess their welfare. Initially, each rabbit underwent surgery in the right eye and the left eye was then operated on two to three weeks after the right eye had recovered. 

The corneal epithelium inside a 9-mm corneal trephine circular mark was scraped off with an ophthalmic blade as previously described [1], without the limbal area being involved. Postoperatively, until the epithelial closure was complete, every rabbit was treated twice a day with topical dexamethasone and chloramphenicol (Deicol^®^ ophthalmic ointment, Alcon laboratories, Barcelona, Spain), diclofenac drops (Voltaren^®^ drops, Allergan, Irvine, CA, OSA), and with one of the treatments under study. Rabbits were randomized for each surgical intervention into one of the following four groups: (1) PBS (control), (2) 90% s-PRGF, (3) 90% s-PRGF and 0.2% NaHA, and (4) 0.2% NaHA. PBS was used for the dilution of treatments. In addition, the order in which the animals in the different experimental groups were treated was randomized.

To assess the size of the residual epithelial defect, the eyes were photographed with and without fluorescein once a day, with a ruler placed in the same plane as the ocular surface, and always at the same time of day. Wounded areas were measured using ImageJ software and results were expressed as mean wound area ± SD in mm^2^. Rabbit eyes were also examined for signs of corneal inflammation and neovascularization.

### 4.10. Immunocytochemistry and Histochemical Analysis

The positive staining for corneal epithelial markers of primary cultures was confirmed by immunolabeling for CK3, CK15, and vimentin markers. Single cells were spin onto microscope slides using a cytospin (Cytofuge; Fisher Scientific, Houston, TX, USA). Cells were fixed with 2% paraformaldehyde (Table S2) and immunostaining was performed in the same manner as with tissue sections (see below).

After both eyes of each animal had been operated on and followed-up; that is, 7 days after surgery for the left eyes and 30 days after surgery for the right eyes, half of the corneas from each treatment were fixed in 2% paraformaldehyde and posteriorly included in paraffin to perform H-E staining. Tissue sections were observed with a phase contrast microscope (Nikon Eclipse TS 100) and images were acquired with the ProgRes CapturePro 2.6 software. We evaluated the structural integrity and histological characteristics of the cornea, as well as the regeneration of the epithelium and cell infiltration. 

The other half of the corneas from each treatment were included in OCT (Optimal Cutting Temperature) compound (TissueTek^®^, Sakura Finetek, NL) and frozen below −80 °C. Tissue sections of 10 μm were made with a Leica CM 3050S cryostat (Leica Biosystems, Barcelona, Spain) and stored below −20 °C until immunofluorescent staining, according to conventional protocols. Briefly, sections were fixed using 2% paraformaldehyde or acetone (Table S2) and permeabilized in the former case with phosphate-buffered saline (PBS) solution containing 0.5% Triton X-100 (Sigma) for 10 min. To minimize nonspecific signals, sections were incubated for an additional hour with blocking solution, consisting of PBS containing 0.1% Triton X-100 (PBT) with 5% BSA and 10% FBS. After that, sections were incubated at 4 °C overnight with the appropriate primary antibodies at the respective dilutions in blocking solution (Table S2). After the sections were washed with phosphate-buffered saline, the samples were incubated with Alexa Fluor secondary antibodies (Invitrogen) (Table S2) for 1 h at room temperature and protected from light. The DNA specific dye DAPI (1 μg/mL, Sigma) was used to detect nuclei. Finally, the sections were mounted with Fluoromount-g (Electron Microscopy Sciences, Hatfield, PA, USA) and photographed with a fluorescence microscope (Zeiss, Göttingen, Germany). 

### 4.11. Statistical Analysis

R program, version 3.4.0. (R Foundation for Statistical Computing, General Public License, University of Auckland, New Zealand) was used to calculate means and standard deviations and to perform statistical tests. To assess the statistical significance of two mean differences we used the Wilcoxon rank sum test. For the statistical comparison of mean differences between treatments we used the Kruskall–Wallis test and Dunn’s test with Bonferroni correction to Multiple Comparisons. For qualitative variables we used the Chi-squared test and Fisher’s exact test. The Kaplan–Meier estimator was also used to study the number of days that corneal epithelial defects took to heal completely. Differences were considered statistically significant when *p*-values were <0.05. 

## 5. Conclusions

In summary, we must bear in mind that the use of biopolymers associated with medications in the eye has its limitations, since they can produce blurred vision after instillation for longer than usual [62,63]. In addition, we have not found additional benefit in terms of in vivo corneal epithelial wound healing when used in combination. Therefore, it is possible that the HA used in the concentrations employed in clinical practice (0.1–0.4%) creates a shield that restricts the contact of the s-PRGF components with their receptors [25], or acts by seizing growth factors, instead of as a vehicle that facilitates long-standing contact of growth factors with the ocular surface, making their combined use have a worse effect than their use alone.

It is critical when evaluating the results of the combination of HA and PRP to perform different tests in which different molecular weights and concentrations of the HA are studied (our HA has 200–400 kDa) with different concentrations and formulations of the PRP it is combined with [64]. In this sense, discrepancies are again found among authors who suggest that L-PRP (leukocyte-rich PRP) has more anti-inflammatory and anabolic effects [65] and others that affirm the opposite [66].

On the other hand, HA is a necessary component of the limbal niche. HA has a very significant role in the maintenance of the phenotype of the limbal progenitor cells [52], so damage to the HA that forms the limbal niche produces an alteration in epithelial corneal regeneration. Similarly, PRP also seems to maintain the undifferentiated phenotype of mesenchymal stem cells [67,68].

In addition, the combination of PRP and HA seems to favor the viability and proliferation of mesenchymal stem cells [69]. However, it remains to be demonstrated that the combination of PRP with HA is superior to the use of each of the treatments separately in the maintenance of the undifferentiated phenotype of the corneal scleral limbal cells.

## Figures and Tables

**Table 1:** In vivo experiment assessing the progression of epithelial wound healing in rabbit eyes treated with s-PRGF, s-PRGF and NaHA, NaHA, and PBS as the control. The results are expressed as mean wound area ± standard deviation in mm^2^.

Treatment	TIME (days)						
	Day 0	Day 1	Day 1.5	Day 2	Day 2.5	Day 3	Day 3.5
**Control**	72.32 ± 7.32	47.02 ± 4.84	33.28 ± 3.06	12.93 ± 3.36	6.12 ± 3.24	0.53 ± 0.99	0.06 ± 0.16
**s-PRGF**	71.91 ± 4.39	46.19 ± 4.06	30.22 ± 4.09	10.00 ± 3.15	3.65 ± 2.79	0.05 ± 0.10	0
**NaHA**	72.48 ± 5.13	45.32 ± 4.20	36.72 ± 7.74	12.46 ± 5.17	5.98 ± 3.69	0.65 ± 1.23	0.10 ± 0.27
**s-PRGF + NaHA**	72.57 ± 2.77	48.06 ± 8.97	37.44 ± 7.63	13.59 ± 5.26	6.14 ± 3.71	0.46 ± 0.76	0.02 ± 0.06

No significant differences between any treatments were found (Kruskal–Wallis test, *p* ≥ 0.05).

**Table 2:** Number of Ki67 positive cells in rabbit corneas treated with s-PRGF, s-PRGF and NaHA, NaHA, and PBS as the control. The results are expressed as mean number of cells ± standard deviation in seven different areas of the central corneas at 7 and 30 days after surgery.

	Treatment			
7 Days	PBS	s-PRGF	s-PRGF + NaHA	NaHA
Epithelium	17 ± 10	4 ± 2	16 ± 15	43 *** ± 20
Stroma	2 ± 1	1 ± 1	2 ± 1	5 ± 4
30 days	PBS	s-PRGF	s-PRGF + NaHA	NaHA
Epithelium	12 ± 6	5 ^Φ^ ± 2	7 ± 3	4 ^ΦΦ^ ± 2
Stroma	0.3 ± 0.5	0.3 ± 0.5	0.3 ± 0.5	0.4 ± 0.8

Statistically significant differences with respect to s-PRGF (*) or PBS (Φ) (^Φ^ *p* < 0.05; ^ΦΦ^ *p* < 0,01; *** *p* < 0.001, Kruskal–Wallis test, Dunn test with Bonferroni correction to Multiple Comparisons).

## Supplementary Materials

Supplementary materials can be found at https://www.mdpi.com/1422-0067/20/7/1655/s1.

## Abbreviations

HA	Hyaluronic acid
HaNA	Sodium hyaluronate
PRP	Platelet Rich Plasma
s-PRGF	Serum derived from Plasma Rich in Growth Factors
HCE	Human Corneal Epithelial
RPCE	Rabbit Primary Corneal Epithelial
PBS	Phosphate Buffered Saline
FBS	Fetal Bovine Serum
BSA	Bovine Serum Albumin
OCT	Optimal Cutting Temperature compound
MTT	3-(4,5-dimethylthiazol-2-yl)-2,5 diphenyltetrazolium bromide
DMSO	Dimethyl Sulfoxide
DAPI	4′,6-diamidino-2-phenylindole
W-H	Wound-Healing
CD	Cluster of Differentiation
CK	Cytokeratin
ZO-1	Zonula Occludens-1
α-SMA	Alpha-Smooth Muscle Actin
