# Supplementary material for: Hyaluronic Acid Combined with Serum Rich in Growth Factors in Corneal Epithelial Defects
Source: Int J Mol Sci. 2019 Apr 3;20(7):1655. doi: 10.3390/ijms20071655 (PMC6480555; doi:10.3390/ijms20071655)
Supplement: Supplementary file 1 [file ijms-20-01655-s001.pdf]

Table S1. Treatments for the culture of RPCE and HCE cells for in vitro assays.

Treatments	Components
BSA-synchronizer	DMEM:F12 + 2 mM L-Glutamine + 1% Penicillin/Streptomycin + 1% BSA
1% BSA (negative control)	DMEM:F12 + 2 mM L-Glutamine + 1% Penicillin/Streptomycin + Supplements* + 1% BSA
10% FBS medium (positive control)	DMEM:F12 + 2 mM L-Glutamine + 1% Penicillin/Streptomycin + Supplements* + 10% FBS
45% s-PRGF	DMEM:F12 + 2 mM L-Glutamine + 1% Penicillin/Streptomycin + Supplements* + 45% s-PRGF
0.1% NaHA medium	DMEM:F12 + 2 mM L-Glutamine + 1% Penicillin/Streptomycin + Supplements* + 0.1% NaHA

* Supplements for RPCE cells: 10 ng/mL EGF + 5 μL/mL insulin + 0.1 μL/mL cholera toxin. Supplements for HCE cells: 10 ng/mL EGF + 5 μL/mL Insulin + 0.1 μL/mL cholera toxin + 0.5% DMSO.

Table S2. Immunohistochemical analysis conditions.

Marker	Commercial company	Dilution	Fixation	Permeabilization	Secondary antibody
**Cytokeratin k3/k76**	Millipore (Ref: CBL218)	1:50	2% PAF	PBS + 0.5% Triton	Alexa Fluor 568 goat anti-mouse IgG1 (Ref: A21124)
**Cytokeratin k15**	Santa Cruz (Ref: sc-47697)	1:50	2% PAF	PBS + 0.5% Triton	Alexa Fluor 488 goat anti-mouse IgG2a (Ref: A21131)
**Alpha smooth muscle actin (α SMA)**	Abcam (Ref: ab7817)	1:400	2% PAF	PBS + 0.5% Triton	Alexa Fluor 488 goat anti-mouse IgG2a (Ref: A21131)
**Integrin beta 4**	Abcam (Ref: ab29042)	1:20	Acetone	NO	Alexa Fluor 568 goat anti-mouse IgG1 (Ref: A21124)
**Ki-67**	Millipore (Ref: MAB4190)	1:20	Acetone	NO	Alexa Fluor 568 goat anti-mouse IgG1 (Ref: A21124)
**ZO-1**	Abcam (Ref: ab190085)	1:20	Acetone	NO	Alexa Fluor 568 donkey anti-goat IgG (Ref: A11057)
**Vimentin**	Abcam (Ref: ab16700)	1:1000	2% PAF	PBS + 0.5% Triton	Alexa Fluor 488 goat anti-rabbit IgG (Ref: A11070

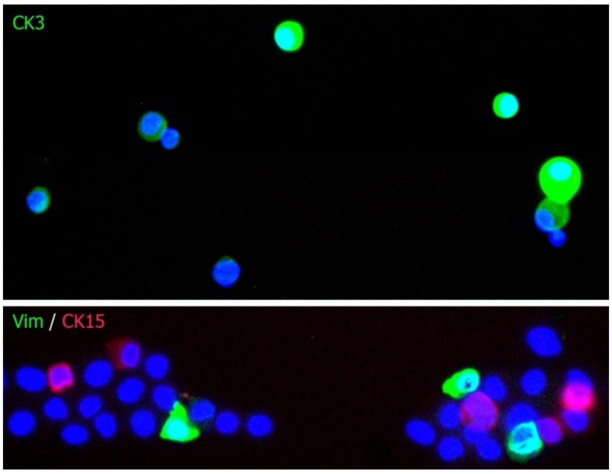

Figure S1. Fluorescent immunostaining of primary cultures. Cells were positive for the CK3 corneal epithelial and the CK15 corneal epithelial progenitor markers. Some cells were positive for the vimentin marker, which stains stromal mesenchymal cells as well as stem/progenitor epithelial corneal cells. Magnification 200×.
